# RGB Color Calibration for Quantitative Image Analysis: The “3D Thin-Plate Spline” Warping Approach

**DOI:** 10.3390/s120607063

**Published:** 2012-05-29

**Authors:** Paolo Menesatti, Claudio Angelini, Federico Pallottino, Francesca Antonucci, Jacopo Aguzzi, Corrado Costa

**Affiliations:** 1 Agricultural Engineering Research Unit of the Agricultural Research Council (CRA-ING), Via della Pascolare 16, Monterotondo scalo (Rome) 00015, Italy; E-Mails: paolo.menesatti@entecra.it (P.M.); oppela@tin.it (C.A.); fedepall@yahoo.it (F.P.); francescaantonucci@hotmail.it (F.A.); 2 Instituto de Ciencías del Mar (ICM-CSIC), Paseo Marítimo de la Barceloneta 37-49, Barcelona 08003, Spain; E-Mail: jaguzzi@cmima.csic.es

**Keywords:** image analysis, color calibration, thin-plate spline, color space

## Abstract

In the last years the need to numerically define color by its coordinates in n-dimensional space has increased strongly. Colorimetric calibration is fundamental in food processing and other biological disciplines to quantitatively compare samples' color during workflow with many devices. Several software programmes are available to perform standardized colorimetric procedures, but they are often too imprecise for scientific purposes. In this study, we applied the Thin-Plate Spline interpolation algorithm to calibrate colours in sRGB space (the corresponding Matlab code is reported in the Appendix). This was compared with other two approaches. The first is based on a commercial calibration system (ProfileMaker) and the second on a Partial Least Square analysis. Moreover, to explore device variability and resolution two different cameras were adopted and for each sensor, three consecutive pictures were acquired under four different light conditions. According to our results, the Thin-Plate Spline approach reported a very high efficiency of calibration allowing the possibility to create a revolution in the in-field applicative context of colour quantification not only in food sciences, but also in other biological disciplines. These results are of great importance for scientific color evaluation when lighting conditions are not controlled. Moreover, it allows the use of low cost instruments while still returning scientifically sound quantitative data.

## 1. Introduction

Color is defined as the visible electromagnetic spectrum reflected by an object and perceivable by a sensor within its detection range, being one of the most important attributes of objects' appearance. Being a highly informative variable, trials for its quantification by measurement (*i.e.*, colorimetry) have conducted since the early 1900s. In fact, the first colorimetric standard CIE L*a*b* was developed in 1931 on the basis of measures under variable daylight, object and human perceptor (*i.e.*, the tristimulus approach) [1,2]. Following the technological evolution together with the development of the Information Communication Technology (ICT), the introduction of the digital imaging widened both the acquisition tools and the rendering media, requiring the elaboration of up-to-date and different color spaces [2,3]. In this new instrumental and applicative context, there was the need to numerically define the colors by their coordinates in n-dimensional space. Although the spectrum is continuous with no clear boundaries between one color and the next ones, color ranges were established as an approximation for coordinate definition [4].

Each color depends on the type of emission source that irradiates an object, as well as on the physical properties of the object itself (which reflects the radiation consequently detected by the sensor), and finally on the in-between medium (e.g., air or water) [5]. Generally, the color spaces applied in product classification are the standard RGB (sRGB; red, green, blue) and L*a*b*. sRGB can be obtained rapidly using computer vision systems. Outputs signals are generated by the camera sensors (e.g., CCD or CMOS), but the rendering is device-dependent, since the range of colors varies according to the display device specifications [2,6,7]. In order to overcome this problem, sRGB values are often transformed to other color spaces such L*a*b*. Moreover, even the result of such transformation is device-dependent [8].

Commonly other sRGB color calibrations, using various methods and algorithms, are used to overcome this problem. References [9,10] evidenced that the extreme lighting conditions of Mars, produced by the peculiar combination in atmospheric dust and the Sun radiation, required a thoughtful color calibration of any acquired image. The same occurs for marine video-imaging, where a calibration is required for the evaluation of animal colouring, according to lighting conditions that vary markedly according to depth [11,12].

Colorimetric calibration prior to photo/image interpretation is gaining increasing attention worldwide in several and diversified disciplines such as biology, since the color is both a phenetic feature of organisms and a feature of the environmental space occupied by organisms [13–15]; food science, where the color is a fundamental property to evaluate quality, freshness and conformity of food products [16–19]; medicine, where, for example, the measurement of color is useful to assess a degree of some pathologies [20]; environmental monitoring, since the Geographic Information System and remote sensing use color, hence requiring its prior calibration for photointerpretation procedures. Also in web applications such as the merceological catalogues, where it can be used as an efficient feature for goods comparisons [21] color calibration results crucial. Of course, robotic video inspection for qualitative and pattern recognition also requires the gathering of precise color information [22].

Many factors influence the chromatic outcome and this could represent either a character to be studied or a source of variability to be adjusted. The color of a natural (animal/food) object can change according to: (i) lifetime [23], shelf-life [24], harvesting conditions [25], or in the case of organisms, according to gender-, age-, or other intra-specific differences [26]; (ii) the photoreceptor or processing system, since different individuals (or devices) can be sensitive to different wavelengths, as in the case of the color blindness; (iii) the processing system (*i.e.*, the interpreter of stimuli coming from the visual receptors), which can exert a distortion reporting a different color from the physically reflected one, as in the case of the well known phenomenon of the “color constancy” in humans, (*i.e.*, the tendency to perceive the same color of an object under different illuminations) [27]; (iv) the lighting condition source. In particular, this latter factor should be deeply considered prior to any colorimetric measurement, since when not properly evaluated it could produce important biases [28]. A possible way to reduce or avoid illumination biases is then represented by taking pictures under standardized light conditions. Nevertheless, this is a difficult condition to satisfy, not only in the field but even in the laboratory.

Several free- or licensed-softwares for digital photography are available and allow a color calibration up to a degree useful for color flow management within a photographic context, using a color profile assignable to the image that deals with different devices (e.g., ProfileMaker, Monaco Profiler, EZcolor, i1Extreme and many others). All these applications work according to the International Color Consortium (ICC) directives: a color profile represents a set of data that characterizes a color within a space [29]. However, the calibration operated throughout these applications is often imprecise in relation to the scientific needs for colorimetric quantification [30]. A colorimetric calibration is therefore often carried out by combining different polynomial algorithms, multivariate statistics, and neural networks approaches [20]. All of these procedures can successfully reduce the external noise to different extents, pointing out that both the camera settings and its sensor's response to light play a crucial role for objective color quantification.

In this context, the present study introduces a novel colorimetric calibration approach that aims to minimize the effects of the illuminants, camera characteristics and settings. Color image calibration was implemented according to a novel approach: the 3-dimensional sRGB Thin-Plate Spline interpolation (TPS-3D). The calibration efficiency of this method was compared with the one obtained through the use of a widely used commercial software (*i.e.*, ProfileMaker) as well as with that obtained by multivariate linear regression (Partial Least Squares).

## 2. Experimental Section

The images utilized in this study come from four markedly different operative field and laboratory contexts, in which different devices and lighting conditions occurred or were artificially created.

### 2.1. Calibration/Validation Setup Experiment

In order to explore the device variability and resolution, two different cameras were used: (i) a commercial high resolution compact Nikon Coolpix P6000, (13.5 real MP-CCD 4.67× sensor) with optical 4× NIKKOR lens, providing TIFF 8 bit images (from NRG RAW format) with good macro features (manual white balance control, exposure and metering methods were enabled); (ii) aprofessional high resolution reflex Canon 30D (with a 8.2 real MP CMOS 1.6× APS-C sensor) with a Canon 10–22 mm f/3.5–4.5 ens (used at 22 mm, equivalent to 35.2 mm on a full-frame sensor) providing TIFF 8 bit images (from CR2 RAW format). For both devices, white balance, metering method and exposure were manually defined, while ISO sensitivity was set to the minimum.

For each sensor, three consecutive pictures were acquired under four different light conditions: (i) 200 watt Tungsten bulbs (5,000° K) (T); (ii) 200 watt weakened Tungsten bulbs plus neon tubes plus environmental light (wTNE); (iii) neon tubes plus environmental light (NE); (iv) full sun (*i.e.*, at midday; 6,500° K) (S). Pictures for the color calibration setup have been taken with three inside altogether different color checkers: the GretagMacbeth ColorChecker SG 140 color-patches, the GretagMacbeth ColorChecker 24 color-patches and the IFRAO Standard ColorChecker 7 color-patches.

### 2.2. Creation of Colorimetric Standard of Reference

**Figure 1.**
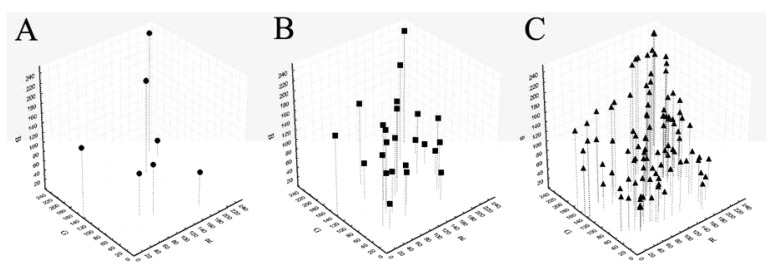
sRGB values of the patches of each ColorChecker in the sRGB space. (**A**) IFRAO Standard ColorChecker 7 color-patches; (**B**) GretagMacbeth ColorChecker 24 color-patches; (**C**) GretagMacbeth ColorChecker SG 140 color-patches.

Being the sRGB and spectral values provided only for the GretagMacbeth ColorChecker 24 color-patches, we preliminarily tested the possibility to calculate reference sRGB values for the other ColorCheckers using spectral values. For each patch of each ColorChecker the spectral reflectance values from 400 to 700 nm (step 10) were extracted using a portable integrated-sphere D50/2 spectrocolorimeter (Xrite, SP64, Grandville, MI, USA). The spectral reflectance values were converted in sRGB [31] using the Matlab OptProp freeware toolbox. Those measured values were considered as reference values for the following calibration treatments. The sRGB values of the patches of each ColorChecker in the sRGB space are reported in Figure 1.

### 2.3. Color Calibration

Each image was calibrated using each of the three ColorCheckers returning three different images. Three different color calibration post-acquisition treatments were used. The first one is the commercial calibration system using the software ProfileMaker Pro 5.0 (PROM). It does not allow the calibration with the IFRAO Standard ColorChecker 7 color-patches, this standard being not present in the software ColorChecker library, or under wTNE and NE because these kinds of illuminants are custom and therefore need professional light meter to be measured and correctly applied in the program, thus losing ease of use for in-field application. The second calibration system is based on a Partial Least Square analysis (PLS) [3,32] and was formerly adopted by Costa *et al.* [19,33] (it is performed with the PLS Toolbox for Matlab 7.1 R14). The innovative third approach is based on a three-dimensional Thin-Plate Spline (TPS-3D) warping, and it will be introduced in detail below.

### 2.4. The TPS-3D Warping Approach

The problem of fitting data in high dimensions arises in a number of applications including data mining, 3D reconstruction of geometric models, fingerprint matching, image warping, medical image analysis and optic flow computations [34]. Warping an image is a transformation which involves pixels mapping from source positions to other destination positions [35]. A commonly used technique to fit the data is the TPS method, which is useful due to its insensitivity to data noise and its capability to minimize the bending energy of a thin-shell object [36]. The name thin plate spline refers to a physical analogy involving the bending of a thin sheet of metal. In the physical setting, the deflection is in the z direction, orthogonal to the plane. In order to apply this idea to the problem of coordinate transformation, one interprets the lifting of the plate as a displacement of the x or y coordinates within the plane. In 2D cases, given a set of K corresponding points, the TPS warp is described by 2(K + 3) parameters, which include six global affine motion parameters and 2K coefficients for correspondences of the control points [37]. These parameters are computed by solving a linear system, in other words, TPS has a closed-form solution. Only a slight modification is necessary to produce interpolation functions for three-dimensional thin-plate splines [38]. Given two configurations of homologous landmarks, the thin-plate spline is a map from plane to plane that maps each landmark to its correspondent. It can be defined briefly, although not quite rigorously, as the interpolation that has the least bending energy, where bending energy is defined to be the integral of the sum of squared second derivatives. Bending energy is zero precisely when the map is affine [39].

In the present work the measured ColorChecker sRGB coordinates within each image (*i.e.*, considering its whole field) were warped (transformed) into the reference coordinates of the same ColorChecker. This transformation was performed through the TPS interpolation function, modified for the three-dimensional space. The three-dimensional sRGB color space is an additive color model in which red, green, and blue light are added together in various ways to reproduce a broad array of colors [40]. The procedure was elaborated in Matlab 7.1 R14 modifying the 2-d TPS code by Ossadtchi [41]. This code refers to Bookstein TPS algorithm. The TPS3D Matlab code is reported in the Appendix. This code, given the set of measured ColorChecker RGB coordinates within the image and the reference coordinates of the same ColorChecker, transforms the RGB value of each pixel of the image following the TPS-3D interpolating function. The code in the Appendix could also be applied to warp 3D images (such as x, y, z references or hyperspectral images) by substituting the colorimetric coordinates with 3D space coordinates.

The TPS algorithm estimates the random data from two paring sets of data to construct the spline map from the affine factor (A) for the linear distortion and weighting factor (W) for the non-linear distortion. The first step of TPS is to solve the following equation (E1) in order to calculate both A and W:

(E1) $$\left[\begin{array}{c} W\\ A \end{array}\right]=\frac{\left[\begin{array}{c} V\\ O\left(4,3\right) \end{array}\right]}{\left[\begin{array}{cc} K & P\\ P^{T} & O\left(4,4\right) \end{array}\right]}$$

where O(r,w) is zero matrix of 4 × 4 and 4 × 3 respectively; P is the matrix of 3d N points (*x, y, z*) to be warped with the additional value 1 in every row (E2) and P^T^ is the transposed P matrix; K is a matrix U(r) for shape distortion by TPS-3D process as defined in E3 and E4 respectively; V is the reference matrix of 3d N points (*x′, y′, z′*) defined in E5:

(E2) $$P=\left[\begin{array}{cccc} 1 & x_{1} & y_{1} & z_{1}\\ 1 & x_{2} & y_{2} & z_{2}\\ \ldots & \ldots & \ldots & \ldots \\ 1 & x_{N} & y_{N} & z_{N} \end{array}\right]$$

(E3) $$K=\left[\begin{array}{ccccc} 0 & U\left(r_{12}\right) & \ldots & U_{1\left(N-1\right)} & U\left(r_{1N}\right)\\ U\left(r_{21}\right) & 0 & \ldots & U_{2\left(N-1\right)} & U\left(r_{2N}\right)\\ \ldots & \ldots & \ldots & \ldots & \ldots \\ U\left(r_{\left(N-1\right)1}\right) & U\left(r_{\left(N-1\right)2}\right) & \ldots & 0 & U\left(r_{\left(N-1\right)N}\right)\\ U\left(r_{N1}\right) & U\left(r_{N2}\right) & \ldots & U\left(r_{N\left(N-1\right)}\right) & 0 \end{array}\right]$$

where, for example:

(E4) $$U\left(r_{12}\right)=2{\left(r_{12}\right)}^{2}\log\left(r_{12}+1E-20\right)$$

where $r_{1,2}=\sqrt{{\left(x_{2}-x_{1}\right)}^{2}+{\left(y_{2}-y_{1}\right)}^{2}+{\left(z_{2}-z_{1}\right)}^{2}}$

(E5) $$V=\left[\begin{array}{ccc} x_{1}^{'} & y_{1}^{'} & z_{1}^{'}\\ \ldots & \ldots & \ldots \\ x_{N}^{'} & y_{N}^{'} & z_{N}^{'} \end{array}\right]$$

### 2.5. Statistical Analyses

In order to quantify the efficiency of the three color calibration techniques, while taking into account the two recording sensors, the three ColorChecker, and the four illumination conditions, mean within-, inter-distances and distance from the reference (ΔRGB; E6) were calculated. This was done also for the non-calibrated images (factor coded as NONE):

(E6) $$\Delta \text{RGB}=\sqrt{{\left(R_{\text{ref}}-R_{i}\right)}^{2}+{\left(G_{\text{ref}}-G_{i}\right)}^{2}+{\left(B_{\text{ref}}-B_{i}\right)}^{2}}$$

Within-distance represents the difference among the sRGB pixel values of the same patches used for the calibration in relation to the reference sRGB values (E7):

(E7) $$\text{Within}-\text{distance}=\frac{\overset{N}{\underset{i=1}{\text{∑}}}\Delta RGB_{i}}{N}$$

where N is the number of patches used for the calibration.

Inter-distances represent differences among mean sRGB pixel values of the same sensor at different illumination conditions of the patches not used for the calibration (E8):

(E8) $$\text{Inter}-\text{distance}=\frac{\overset{M}{\underset{j=1}{\text{∑}}}\Delta RGB_{j}}{M}$$

where M is the number of patches not used for the calibration.

Nested ANOVAs have been used to test if the results, obtained by the different calibration methods (including NONE) and expressed as ΔRGB, were statistically different, and how the difference was due to the nested factors camera, light setting and ColorChecker. The Tukey HSD test for unequal sample size has been used in order to evaluate the homogeneity of the results among calibrations and within them, when considering the nest factors. In order to show how the outputs from the calibration methods depart from the reference point, a Principal Component Analysis (PCA) was performed on the calibration setup experiment dataset (*i.e.*, color coordinates), based on the ANOVA's results (see below). Only the graphs obtained from calibrations based on the GretagMacbeth ColorChecker 24 color-patches were shown. Being the TPS-3D an interpolation method, the null hypothesis of independence between the minimum distance of a calibrated patch not used to calibrate the image and a calibrated one (*i.e.*, between a patch and the similar one in the ColorChecker used to calibrate the image) and the ΔRGB value (*i.e.*, the distance of the calibrated patch from the reference) was tested on the GretagMacbeth ColorChecker 24 color-patches using the Spearman correlation test.

## 3. Results

**Figure 2.**
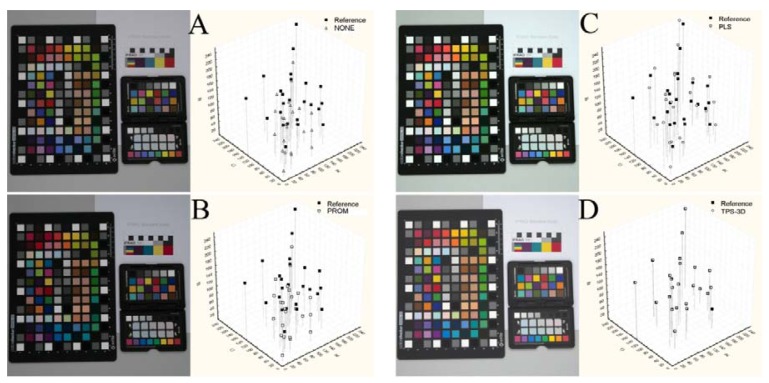
On the left side of the Figure, the original (NONE) image (**A**) and post-calibration with the GretagMacbeth ColorChecker 24 color-patches images (**B**: PROM; **C**: PLS; **D**: TPS-3D) illuminated with 200 watt Tungsten bulbs (5,000° K). On the right side of the Figure, the correspondent reference (black squares) and pre-/post-calibration (white symbols) RGB values based on the GretagMacbeth ColorChecker 24 color-patches (within-distance —Table 1).

Figure 2 reports an example of images in their original status (NONE) and calibrated (using the GretagMacbeth ColorChecker 24 color-patches) with the different methods. On the right side of the same image was reported the reference and pre-/post-calibration (white symbols) sRGB values of the GretagMacbeth ColorChecker 24 color-patches on the sRGB color space (see also Figure 1).

The differences between the Reference coordinates and the pre-/post-calibration ones represent the within-distances (Table 1). The distances from the color references were significantly different among the calibration methods (Table 1; F_3,215_ = 1,723.8, p < 0.001); the *post-hoc* test showed that TPS-3D performed a better performance than PLS. PROM gave results similar to those obtained without any kind of calibration.

The camera type significantly influenced the performance of the calibration methods (F_4,215_ = 1,017.8, p < 0.001), even though TPS-3D seemed to be the less affected compared to the other methods (Table 2).

Also the light settings significantly affected the performance of the calibration methods (F_4,215_ = 44.3, p < 0.001; Table 3), in particular a *post-hoc* test showed that: TPS-3D performed better under LNA and LF than E and NA (four overlapped homogeneousness groups), PLS is the most robust against light variability (two groups), the sensitivity of PROM to light was high (two different groups for the two light conditions handled by this method). The *post-hoc* test for the ColorChecker (F_7,215_ = 24.7, p < 0.001; Table 4) showed that TPS-3D performed better with 24 and 140 patches, whilst PLS and PROM are less, if any, prone to the number of the patches used as reference for the calibration.

The effect of the calibration method on inter-distances resulted to be significantly different too (Table 1; F_2,673_ = 2,374.11, p < 0.001). The *post-hoc* test showed that TPS-3D gave the better performance. Further, even if the number of patches used for the calibration significantly affected the performance of the calibration methods (F_5,673_ = 14.63, p < 0.001; Table 4), a *post-hoc* test showed that TPS3D is not affected by this factor at all, whilst it played a role in the performance of both PLS and PROM. Further, even if the used camera affects the calibration performance (F_3,673_ = 1,142.57, p < 0.001; Table 2), both TPS-3D and PLS were poorly influenced by this factor (two overlapped homogeneousness groups). At the contrary, PROM outputs were deeply affected by the camera factor.

**Figure 3.**
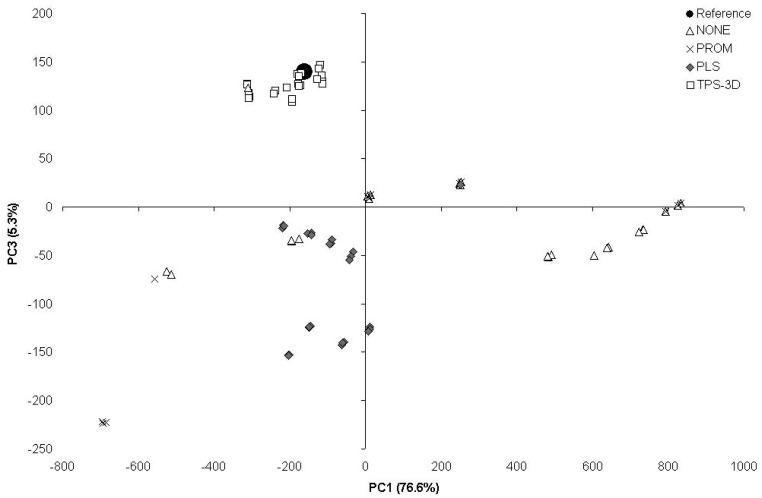
Scatter plot of axis 1 and 3 scores of the PCA based on the scores obtained from the 140- and 7-patches calibrated from the ColorChecker 24-patches.

The PCA based on the scores obtained from the 140- and 7-patches calibrated from the ColorChecker 24-patches (Figure 3) showed that the outputs from TPS-3D clustered close to the reference point. The outputs from PLS were at the same time less clustered and further away from the reference, whilst the outputs from both PROM and NONE resulted to be widely scattered.

The null hypothesis of independence between: (i) the minimum distance of a calibrated patch not used to calibrate the image and a calibrated one (*i.e.*, between a patch and the similar one in the ColorChecker 24-patches used to calibrate the image); and (ii) the ΔRGB value (*i.e.*, the distance of the calibrated patch from the reference) that was tested by the Spearman correlation test was rejected (*r* = 0.41, n = 147, p < 0.001). This indicates that there is an association between the minimum distance from a calibrated patch and the ΔRGB.

## 4. Discussion

Under markedly variable operative conditions we efficiently carried out a sRGB color calibration by using the 3D Thin-Plate Spline warping approach, the efficiency of which was tested not only in comparison with already commercially available protocols (PROM), but also in comparison to another statistical approaches (PLS). While the TPS approach is commonly used in the geometric morphometric framework [37,42] for 2-dimensional and 3-dimensional data [38], we applied it here to a virtual 3-dimensional color space.

In many research fields, the evaluation of color patterns through digital calibrated images is of extreme importance [28]. Anyway, available calibration protocols are based on too rigid approaches. They often use linear models to treat the color within the color space, dealing with each of the RGB channels separately [6,9,20,28]. Being the spectrum continuous, with no clear boundaries between one color and the next one [4], the environment lighting can consistently alter the intensity of the reflected light. Operators should be aware to the attention needed for image acquisition, choosing proper illumination (also in field operations, where possible), camera settings (as proposed by Stevens *et al.* [28] and suggested in the present work) and using appropriate color charts. Camera settings are fundamental to achieve a good color calibration. While a slight over- or under-exposure can be partially recovered if using the RAW camera format mode, metering method, ISO sensitivity and white balance need to be set properly due to the information lost during shooting or to the cameras internal software making changes directly on the image. Moreover, besides camera settings the illumination must be as uniform as possible. The TPS-3D colorimetric calibration consistently reduced this effect given its peculiar way of functioning based on the physical analogy, involving the bending of a thin sheet of metal. This method used color as x, y, z coordinates within a color space. Therefore, from an applicative point of view, color coordinates can be treated with the tools of geometric morphometry (*i.e.*, shape analysis), when color points are considered as equivalent to landmarks. Color points, as landmarks, can be fitted within a consensus configuration [42–44]. According to our results, this kind of fitting has a greater efficiency by far with respect to the commercial methods (ProfileMaker) and the multivariate PLS approach (see Tables 1–4, and Figures 2–3). As a proof of that, the proposed colorimetric calibration method significantly diminished both, distances from the reference and the inter-distances setup experiment. Further, the proposed method results to be the most robust against different lighting conditions and sensor typology, giving the opportunity to use different ColorCheckers. These results are of great importance especially for scientific color evaluation when lighting conditions are not controlled (e.g., in-field studies).

**Figure 4.**
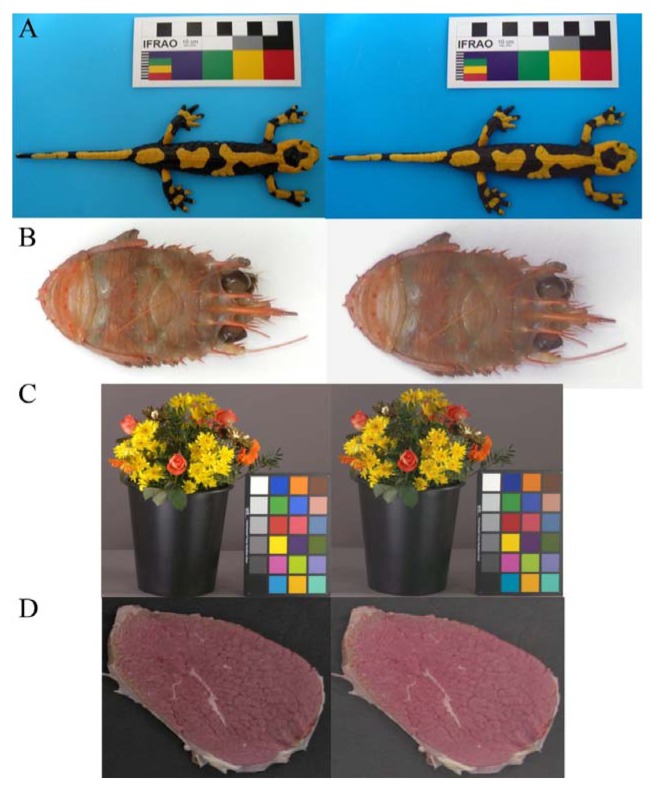
Four example of original (left) images and ones calibrated with the TPS-3D algorithm (right). (**A**) *Salamandra salamandra*; (**B**) *Munida tenuimana*; (**C**) Flower bouquet; (**D**) Beef cutlet.

Color evaluation in biological studies requires the standardization of acquired images, in order to obtain results that do not depend from light conditions and receptors (*i.e.*, the cameras used). This is required not only to assess intra- and inter-individual color differences, but also to study the visual perception [27] of animals and under different light conditions. In Figure 4 four examples of original and TPS-3D calibrated images are reported. With the ever increasing power and availability of digital photography, an increasing number of studies are using image analysis techniques to evaluate the content of color signals [28]. The fire salamander (*Salamandra salamandra*; Figure 4(A)) example points out the possibility to study animals (and plants) diversity with in-field pictures returning information on species, population or sexual variability, since it was shown that these features exert an effect on the color of individuals [45,46]. Color calibration is of great importance also in marine biology; the example of squat lobster (*Munida tenuimana*; Figure 4(B)) could contribute to show different cryptic colorations depending from the selective effect exerted by visual predators (and their optic photoreceptive systems) that uses solar or bioluminescent light to identify their preys. Therefore, the calibration is mandatory prior any quantification of individual differences when populations of different depths or species are considered. These research efforts will eventually be biased if the analyses are carried out on not properly calibrated images. We consider also the importance of color calibration for the commercialization of live goods. In the flower sector one the most important sale channels is the on-line auction [47]; during these processes buyers have to trust in the quality of the flowers (Figure 4(C)), since they could not see the product and have to depend on the computer information [48]. Thus, the guarantee of the application of an optimal color calibration to obtain standardized images will improve customer's trust. Color represents a crucial attribute to be objectively instrumentally measured for a wide range of industrial applications in relation to food processing (e.g., online food sorting into commercial classes, freshness, products shelf-life evaluation [7,49] and the image of a beef cutlet is only an example (Figure 4(D)).

Being the TPS-3D a method based on interpolation, the error (ΔRGB) reduced while increasing the reference points (*i.e.*, from ColorChecker 7-patches to 140-patches). However, the results we obtained suggest that the use of ColorChecker with 24-patches is sufficient to resume the entire color space, while 7-patches are insufficient and 140-patches oversamples, without giving a decisive efficiency increase. Moreover, the error (ΔRGB) resulted significantly greater when the distance of a color from the closer reference ones is greater. This result suggests the use of new ColorCheckers whit 24–30 patches better distributed in the color space. When the colors to be calibrated occupy a limited part of the color space (for example the reds and whites for the cutlets, or the greens for leaves) it is possible to use ColorCheckers which better resume the information of that portion of the color space. From an operative point of view, this approach will allow the use of handmade *ad hoc* ColorCheckers which could be created and printed on a proper paper and simply measured *a posteriori* with a spectrocolorimeter.

The TPS-3D method could be extended to other color spaces (CIE L*a*b*, YUV, HSV, CMYK and, *etc.*) operating from a three-dimensional base (*i.e.*, the RGB color space) to an n-dimensional multi-hyperspectral space [3,50]. The coupling of this method with warping techniques, drawn from geometric morphometry [3,51], allows the superimposition, pixel per pixel, of comparable objects such as animals [3,51,52], fruits [53] and food [54].

## 5. Conclusions

In light of the reported results the TPS-3D method could revolutionize image analysis, due to the wide possible applications available and in thinking and approaching environmental measurements. Moreover, such a method allows the use of low cost instruments (*i.e.*, commercial cameras) while returning quantitative scientifically sound data.

## Figures and Tables

**Table 1.** Results of the Calibration Experiments: Mean ± Standard Error (SE) of the calculated distances (see Material and Methods section for further details).

	**Distances from reference**	**Within-distances**	**Inter-distances**

**NONE**	30.05 ± 1.48	55.76 ± 2.39	-
**PROM**	30.71 ± 2.75	57.65 ± 2.87	44.6 ± 4.84
**PLS**	16.01 ± 0.37	28.33 ± 0.74	10.67 ± 0.19
**TPS-3D**	10.39 ± 0.41	11.11 ± 0.51	9.55 ± 0.16

**Table 2.** Results of the Calibration Experiments (Sensors): values refer to the performance of the calibration methods taking into account the kind of device used for the taking the pictures (see Table 1 for codes).

	**Device**	**Distances from reference**	**Inter-distances**

**NONE**	Canon	41.71 ± 0.78	-
	Nikon	18.39 ± 0.69	-

**PROM**	Canon	43.2 ± 1.42	73.18 ± 2.2
	Nikon	18.22 ± 1.1	16.01 ± 0.36

**PLS**	Canon	18.55 ± 0.37	10.81 ± 3.84
	Nikon	13.48 ± 0.25	10.52 ± 0.23

**TPS-3D**	Canon	11.08 ± 0.66	9.69 ± 0.23
	Nikon	9.7 ± 0.45	9.42 ± 0.21

**Table 3.** Results of the Calibration Experiments (Light Settings): values refer to the performance of the calibration methods taking into account the light settings (see Table 1 for codes).

	**Light settings**	**Distances from reference mean ± SE**

**NONE**	T	34.43 ± 3.04
	wTNE	29.03 ± 3.58
	NE	32.04 ± 2.26
	S	24.72 ± 2.49

**PROM**	T	34.36 ± 3.80
	wTNE	-
	NE	-
	S	27.06 ± 3.83

**PLS**	T	17.71 ± 0.71
	wTNE	16.17 ± 0.80
	NE	16.52 ± 0.68
	S	13.64 ± 0.47

**TPS-3D**	T	9.6 ± 1.02
	wTNE	8.88 ± 1.00
	NE	11.83 ± 0.46
	S	11.25 ± 0.42

**Table 4.** Results of the Calibration Experiments (ColorCheckers): values refer to the performance of the calibration methods taking into account the ColorChecker (see Table 1 for codes).

	**Color Checker**	**Distances from reference mean ± SE**	**Inter-distances**

**NONE**	7	29.66 ± 2.55	-
	24	29.86 ± 2.58	-
	140	30.64 ± 3.66	-

**PROM**	7	-	-
	24	30.81 ± 3.94	46.28 ± 6.99
	140	30.61 ± 4.00	42.92 ± 6.88

**PLS**	7	16.89 ± 0.72	11.76 ± 0.34
	24	15.42 ± 0.63	11.34 ± 0.33
	140	15.73 ± 0.58	8.9 ± 0.24

**TPS-3D**	7	14.3 ± 0.37	9.52 ± 0.28
	24	8.71 ± 0,44	9.78 ± 0.28
	140	8.16 ± 0.45	9.36 ± 0.25

## Appendix

TPS-3D script to warp a three-dimensional configuration (ZZp) into another configuration (ZZg) using the Thin-Plate Spline interpolating algorithm. The warping implemented will translate ZZp into ZZg exactly.

TPS-3D matlab script implemented the 2D code by Ossadtchi G. (2001), previous named as ‘Simple Warping Routine’ (http://www.mathworks.com/matlabcentral/fileexchange/1203-simple-warping-routine) which was adapted from Bookstein F.L., 1989. Principal Warps: Thin-Plate splines and the decomposition of deformations. IEEE Transactions on Pattern Analysis and Machine Intelligence, 11(6). (request Matlab V7.0 and Statistical Toolbox).

%INPUT VARIABLES

%ZZp, matrix MxN where N is three columns (X,Y,Z or RGB) and M are the points to be warped

%ZZg, matrix MxN where N is three columns (X,Y,Z or RGB) and M are the reference points

NPs = size(ZZp,1);

Xp = ZZp( : ,1) ′ ;

Yp = ZZp( : ,2) ′ ;

Zp = ZZp( : ,3) ′ ;

Xg = ZZg( : ,1) ′ ;

Yg = ZZg( : ,2) ′ ;

Zg = ZZg( : ,3) ′ ;

rXp = repmat(Xp( : ),1,NPs);

rYp = repmat(Yp( : ),1,NPs);

rZp = repmat(Zp( : ),1,NPs);

wR = sqrt((rXp-rXp′).ˆ2 + (rYp-rYp′).ˆ2+ (rZp-rZp′).ˆ2);

wK = 2*(wR.ˆ2).*log(wR+1e-20);

wP = [ones(NPs,1) Xp( : ) Yp( : ) Zp( : )];

wL = [wK wP;wP′ zeros(4,4)];

wY = [Xg( : ) Yg( : ) Zg( : ); zeros(4,3)];

wW = inv(wL)*wY;

% matrix to be warped

X=ZZp( : ,1) ′ ;

Y=ZZp( : ,2) ′ ;

Z=ZZp( : ,3) ′ ;

NWs = length(X);

rX = repmat(X,NPs,1);

rY = repmat(Y,NPs,1);

rZ = repmat(Z,NPs,1);

rXp = repmat(Xp( : ),1,NWs);

rYp = repmat(Yp( : ),1,NWs);

rZp = repmat(Zp( : ),1,NWs);

wR = sqrt((rXp-rX).ˆ2 + (rYp-rY).ˆ2 + (rZp-rZ).ˆ2);

wK = 2*(wR.ˆ2).*log(wR+1e-20);

wP = [ones(NWs,1) X( : ) Y( : ) Z( : )]′ ;

wL = [wK;wP]′ ;

% warped coordinates

Xw = round(wL*wW( : ,1));

Yw = round(wL*wW( : ,2));

Zw = round(wL*wW( : ,3));

All= [ZZp, ZZg, Xw, Yw, Zw].
